# Field Effect Transistor with Nanoporous Gold Electrode

**DOI:** 10.3390/mi14061135

**Published:** 2023-05-28

**Authors:** Ezzat G. Bakhoum, Cheng Zhang

**Affiliations:** 1 Department of Electrical and Computer Engineering, University of West Florida, Pensacola, FL 32514, USA; 2 Department of Mechanical Engineering, University of West Florida, Pensacola, FL 32514, USA; czhang@uwf.edu

**Keywords:** MOSFET, nanoporous gold, bioelectrochemical reactions

## Abstract

Nanoporous gold (NPG) has excellent catalytic activity and has been used in the recent literature on this issue as a sensor in various electrochemical and bioelectrochemical reactions. This paper reports on a new type of metal–oxide–semiconductor field-effect transistor (MOSFET) that utilizes NPG as a gate electrode. Both n-channel and p-channel MOSFETs with NPG gate electrodes have been fabricated. The MOSFETs can be used as sensors and the results of two experiments are reported: the detection of glucose and the detection of carbon monoxide. A detailed comparison of the performance of the new MOSFET to that of the older generation of MOSFETs fitted with zinc oxide gate electrodes is given.

## 1. Introduction

**Figure 1:**
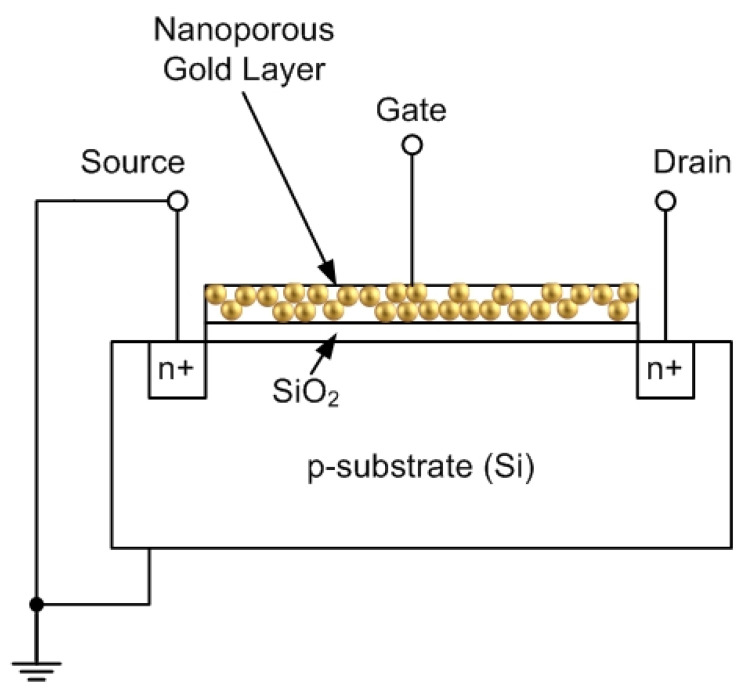
Structure of proposed field effect transistor with nanoporous gold gate electrode.

Nanoporous gold (NPG) has received widespread attention recently because of its excellent catalytic activity. Nanoporous gold has been used to detect numerous analytes in electrochemical, bioelectrochemical, and optical sensing applications [1,2,3,4,5,6,7,8,9,10,11,12,13]. The objective of this paper is to introduce a new type of metal–oxide–semiconductor field-effect transistor (MOSFET) that uses nanoporous gold as a gate electrode. A cross-sectional view of the proposed MOSFET is shown in Figure 1.

Both n-channel and p-channel MOSFETs have been fabricated based on the principle shown in Figure 1 (the total number of devices that were fabricated and tested was 4). The principle of operation of the device shown in Figure 1 is as follows: when an electrochemical or bioelectrochemical reaction occurs inside the pores of the gate electrode, the work function of the metal (in volts) is reduced substantially [14,15,16]. Accordingly, the threshold voltage of the MOSFET is reduced (see Equation (1) in Section 3). This results in an increased drain current $I_{D}$. This increase in the drain current can be detected with external circuitry. Two examples of the detection of analytes are given in this paper: the detection of glucose, and the detection of carbon monoxide.

As a background, NPG was used by Chen et al. [4] and by Li et al. [5] in a 3-electrode system for the detection of glucose. NPG was also used by Wittstock et al. [11] for the detection of methanol and for the detection of carbon monoxide [12] at low temperatures. An optical-based sensor that uses NPG for the detection of mercury was introduced by Zhang et al. [13] and discussed separately by Liu et al. [16]. Several references that describe a MOSFET with a gate electrode that consists of zinc oxide (ZnO) nanowires exist in the literature [17,18,19,20,21,22,23]. That type of MOSFET has been used for the biosensing of glucose, hydrogen peroxide, and enzymes. References [24,25] describe several additional types of MOSFET that utilize materials such as graphene, MoS_2_, and SnS_2_ for the detection of some specific types of analytes.

As far as the authors are aware, this paper introduces for the first time a sensor that consists of a MOSFET that is fitted with an NPG gate electrode. A detailed comparison between the performance of the new MOSFET and the older MOSFET that uses ZnO nanowires is given in Section 5.

## 2. Fabrication/Assembly of the Prototype MOSFETs

**Figure 2:**
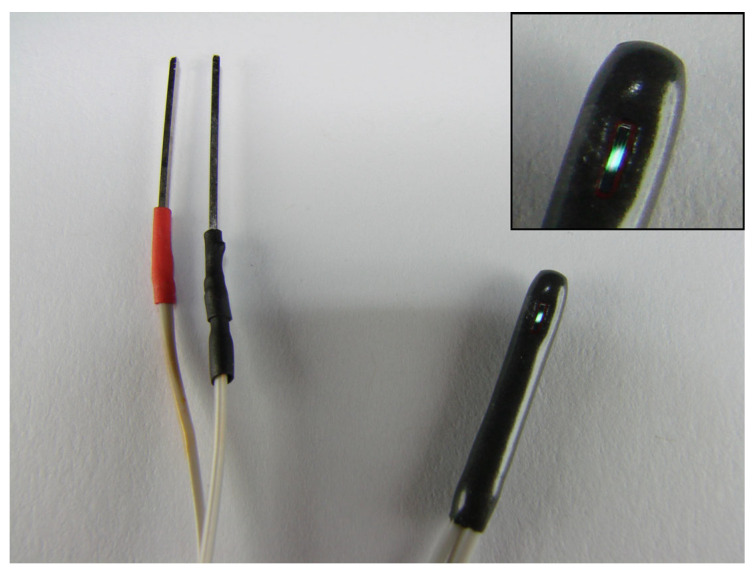
Photograph of the ISFET transistor. The SiO_2_ layer is exposed to the air. The transistor has an exposed gate area of 1 mm × 4 mm (the inset shows an enlarged view of the exposed gate area). The transistor has only two terminals: drain and source.

Ion-sensitive field-effect transistors (ISFET) were obtained from a commercial supplier (Winsense Corporation, Bangkok, Thailand). ISFETs are essentially MOSFETs without gate electrodes or gate terminals. This type of transistor has an open cavity, and the silicon dioxide layer is exposed to the air. The transistor is shown in Figure 2. The open cavity allows for the deposition/insertion of a gate electrode via sputtering.

**Figure 3:**
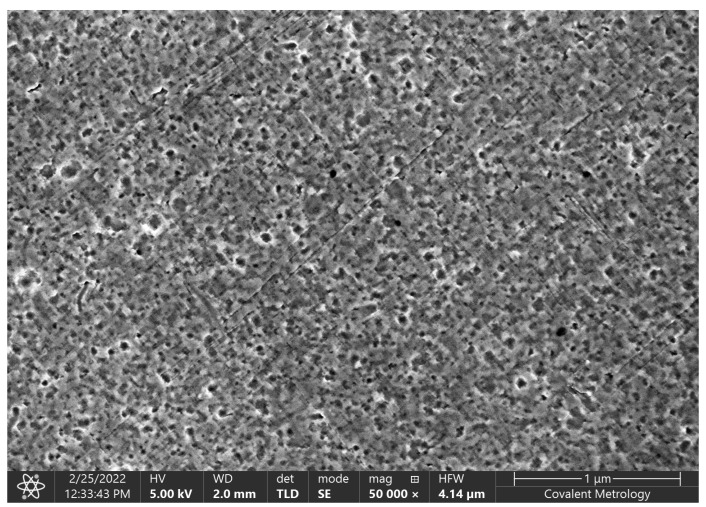
SEM photograph of the gate electrode after soaking in nitric acid for 15 min. The surface is porous, with an average pore size of 20 nm. (Before the soaking procedure, the surface was a smooth metallic surface with no pores.)

Twelve-karat gold leaves were obtained from a number of commercial sources. The 12-karat gold consisted of 50% gold (Au) and 50% silver (Ag). A thin layer of the 12-karat gold was deposited inside the open cavity of the ISFET via sputtering. This step created a gate electrode that consisted of 50% Au and 50% Ag. The thickness of the gate electrode was determined to be approximately 0.5 mm. In order to create an NPG gate electrode, the FETs were soaked in concentrated nitric acid (HNO_3_) for durations that ranged from 15 min to 4 h [3]. The nitric acid selectively dissolved Ag, but not Au. When subjected to a soaking period of 15 min, this step created an NPG electrode with an average pore size of 20 nm. For a soaking period of 4 h, the resulting NPG electrode had an average pore size of 50 nm. Figure 3 shows a scanning electron microscope (SEM) photograph of the surface of the NPG electrode after the step of dissolving the silver in nitric acid.

**Figure 4:**
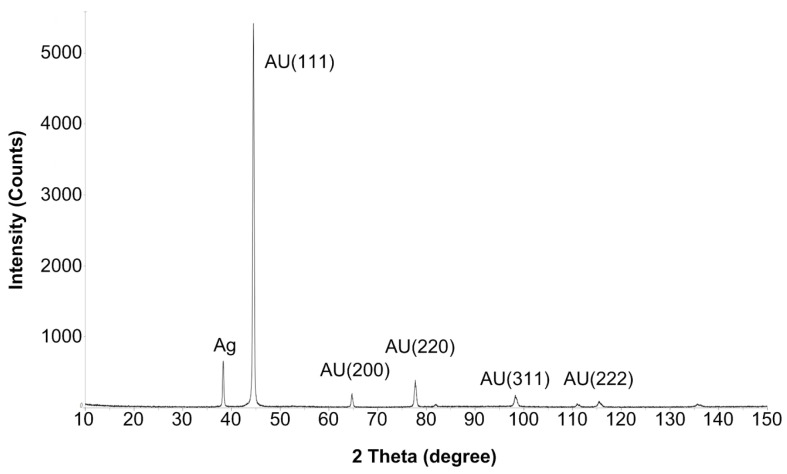
X-ray diffraction spectrum of the surface of the nanoporous gold electrode.

Figure 4 shows an X-ray diffraction spectrum of the surface of the NPG electrode. The figure shows that most of the silver has been removed by etching in nitric acid.

## 3. Theory of the MOSFET with NPG Gate Electrode

The threshold voltage $V_{TH}$ of a MOSFET is given by [26]

(1) $$V_{TH}=\Phi_{M}-\Phi_{si}-\frac{Q_{ox}+Q_{ss}+Q_{B}}{C_{ox}}+2\Phi_{f}$$

where $\Phi_{M}$ is the work function of the gate metal (in volts), $\Phi_{si}$ is the work function of the bulk semiconductor (in volts), $Q_{ox}$ is the accumulated charge in the oxide layer, $Q_{ss}$ is the fixed charge per unit area at the insulator-semiconductor interface, $Q_{B}$ is the semiconductor depletion charge per unit area, $C_{ox}$ is the capacitance of the oxide layer per unit area, and $\Phi_{f}$ is the Fermi potential of the semiconductor.

The drain current $I_{D}$ of the MOSFET in the non-saturated region is given by [26]

(2) $$I_{D}=C_{ox}\mu \frac{w}{l}\left(\left(V_{GS}-V_{TH}\right)V_{DS}-\frac{1}{2}V_{DS}^{2}\right)$$

where μ is the electron mobility, w is the width of the channel, l is the length of the channel, $V_{GS}$ is the gate-source voltage, and $V_{DS}$ is the drain–source voltage. In the saturated region, $I_{D}$ is given by

(3) $$I_{D}=\frac{1}{2}C_{ox}\mu \frac{w}{l}{\left(V_{GS}-V_{TH}\right)}^{2}$$

Equations (1)–(3) show that a reduction in the work function of the gate electrode will result in a reduction in the threshold voltage of the MOSFET, and hence an increase in the drain current.

## 4. Experimental Results

### 4.1. Cyclic Voltammetry for MOSFET in a Glucose Solution

**Figure 5:**
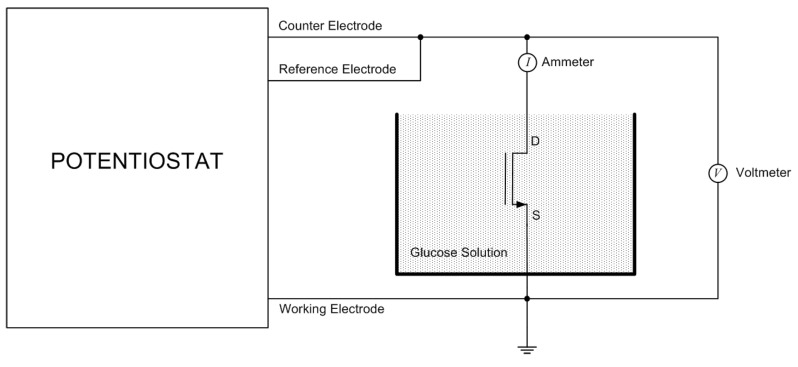
Circuit configuration of a potentiostat controlling the Drain–Source voltage of the MOSFET in a 2-electrode configuration. The MOSFET is immersed in a glucose solution.

**Figure 6:**
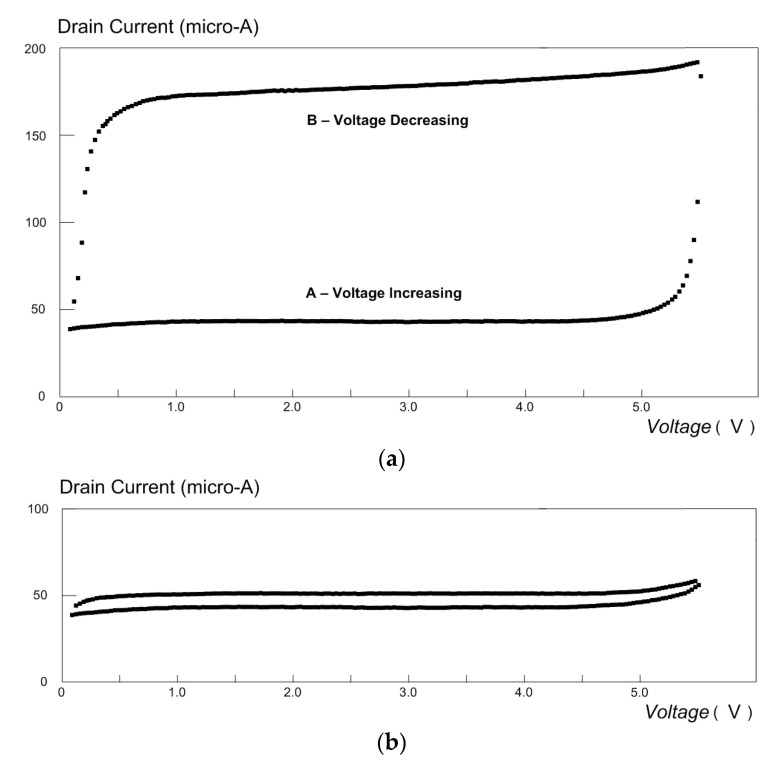

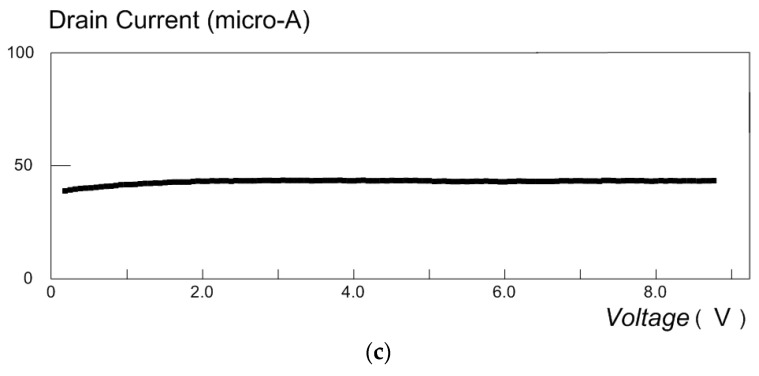
(**a**). Cyclic voltammetry plots for MOSFET in a glucose solution. The drain current increases sharply at $V_{DS}\approx 5.5$ V due to the oxidation of glucose inside the NPG electrode. (Concentration = 50 g/L). (**b**) Cyclic voltammetry plots for MOSFET in a glucose solution. (Concentration = 0.7 g/L). (**c**) Cyclic voltammetry plots repeated with a solution that consists only of PBS.

The MOSFET with the NPG gate electrode can be used as a biosensor for glucose [1,4]. This can be applied in the diagnosis of diabetes. The test set up for the detection of glucose is shown in Figure 5. As shown, the MOSFET (with the NPG layer attached to the gate area) was connected to a potentiostat (Princeton Applied Research, model 363). The experiment was carried out in a two-electrode configuration, as shown in the figure. In this configuration, the potentiostat controlled the voltage between the working electrode and the counter electrode. This voltage is the drain–source voltage ($V_{DS}$) that is applied to the transistor. The drain current ($I_{D}$) is measured with an ammeter, as shown. The MOSFET is immersed in a phosphate buffer solution (PBS, pH ≈ 7.4) in which glucose is dissolved with a concentration of 50 g/L. A cyclic voltammetry scan is shown in Figure 6a.

As shown on segment A of the plot in Figure 6a (the lower segment), a conventional curve for $I_{D}$ is obtained as a function of $V_{DS}$. However, at $V_{DS}\approx$ 5.5 V, the drain current rises sharply. This peak at 5.5 V occurs as a result of the oxidation of glucose inside the pores of the NPG layer [4,5,27,28]. This bioelectrochemical reaction results in a lower work function of the NPG and hence a substantially higher drain current. The cyclic voltammetry scan was repeated with a solution in which the glucose concentration was only 0.7 g/L. The result is shown in Figure 6b. This result shows that the sensor is suitable for the detection of diabetes. Finally, the test was repeated with a solution that that consists only of PBS, and no peak in the current was found (Figure 6c).

**Figure 7:**
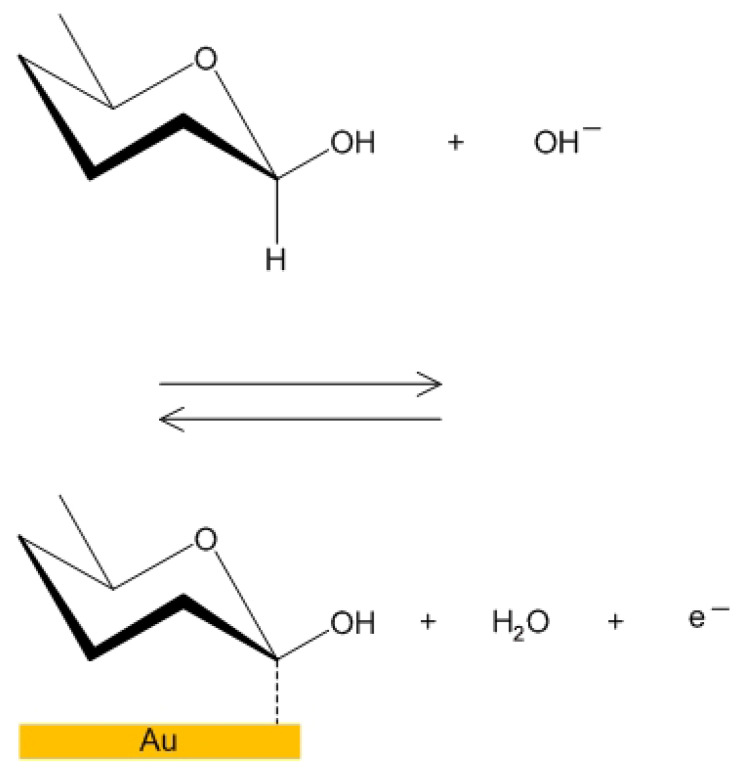
Oxidization of glucose inside NPG (first reaction).

**Figure 8:**
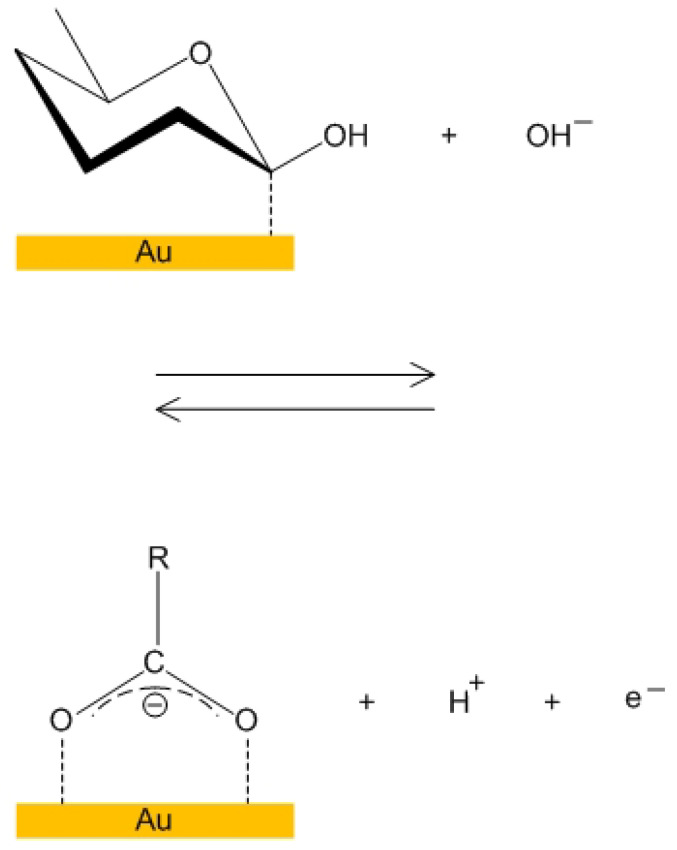
Oxidization of glucose inside NPG (second reaction), which results in the formation of gluconate.

The bioelectrochemical reaction inside the pores of the NPG electrode was studied and by Pasta et al. [28] and the results are illustrated in Figure 7 and Figure 8.

### 4.2. Detection of Carbon Monoxide

A p-channel MOSFET was prepared by depositing 12-karat gold on the gate of a p-channel ISFET and by soaking the ISFET in nitric acid as described previously (which results in a layer of NPG covering the gate area). A gate terminal was added, and the gate terminal was connected to the source terminal (and hence $V_{GS}=0$). The MOSFET was placed in a small stainless-steel chamber, and a mixture of carbon monoxide gas (CO) and air was pumped inside the chamber. The concentration of CO, which was measured with a flowmeter, ranged from 100 ppm to 500 ppm. The following two reactions can occur inside the pores of the NPG layer [29]:

(4) $$2\mathrm{CO}+O_{2}^{-}\to 2{\mathrm{CO}}_{2}+e^{-}\;\mathrm{CO}+O^{-}\to {\mathrm{CO}}_{2}+e^{-}$$

**Figure 9:**
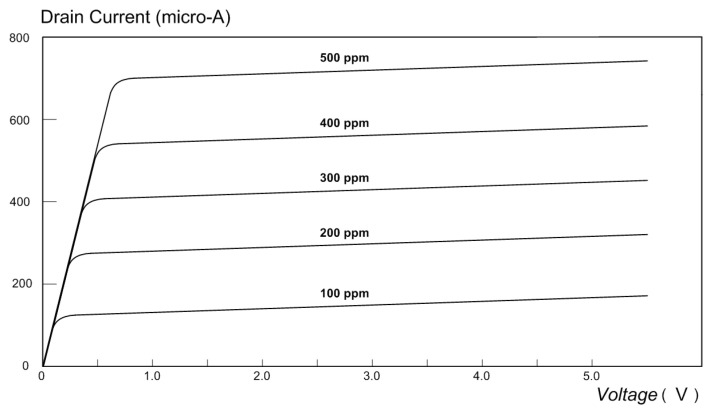
Drain current as a function of the drain–source voltage, for CO concentrations ranging from 100 ppm to 500 ppm.

The release of free electrons inside the NPG layer attracts positive charges in the p-channel and allows the MOSFET to conduct current. Figure 9 shows the measured drain–source current as a function of the drain–source voltage for CO concentrations ranging from 100 ppm to 500 ppm. Clearly, the drain–source current increases as the number of free electrons in the NPG layer increases, i.e., as the concentration of CO increases (note that $V_{GS}=0$, and hence the transistor current is not a function of $V_{GS}$ in this experiment). Hence, the MOSFET with the NPG gate electrode is a sensitive detector of CO gas.

## 5. Discussion and Comparison of the NPG MOSFET to the ZnO MOSFET

To compare the performance of the new NPG MOSFET introduced in this paper to that of the older zinc oxide (ZnO) MOSFET, we consider three points of comparison:

- Deposition of the gate electrode: only atomic layer deposition (ALD) technique is known to be practical for the task of successfully depositing ZnO nanowires as a gate electrode in a MOSFET. ALD, however, is an expensive technique that is not suited for mass production. By comparison, the NPG gate electrode can be easily deposited by sputtering as described earlier. Sputtering is a low-cost technique that is suitable for mass production.
- Leakage currents: The leakage currents in ZnO MOSFETs are known to be high because of the degradation of the oxide layer during the growth process of the ZnO nanowires. The gate-source leakage current, for example, is typically in the order of a few hundred nA. In the present MOSFET, the leakage current is substantially lower because the NPG layer is deposited by sputtering. The gate-source leakage current in the present MOSFET was measured with a Tektronix DMM4020 multimeter and was found to be approximately 1 nA, which is substantially less than the leakage current in ZnO MOSFETs.
- Sensitivity: The sensitivity of a MOSFET that is fitted with a chemically sensitive gate electrode is given by the following equation [30]:
  (5) $$\mathrm{Sensitivity}\;(S)=\frac{\Delta G}{G_{0}}=\frac{2\epsilon_{0}\epsilon_{r}\Phi_{B}N\left(t\right)}{qa^{2}N_{D}\log\left(1+\frac{t_{d}}{a}\right)}$$
  where ΔG is the change in the conductivity of the MOSFET, $G_{0}$ is the initial conductivity, $\epsilon_{0}$ is the permittivity of free space and $\epsilon_{r}$ is the relative permittivity of the oxide layer, $\Phi_{B}$ is the surface potential, $N_{t}$ is the density of charge states at the surface, q is the electron’s charge, a is a geometry dependent parameter, $N_{D}$ is the concentration of donor atoms, and $t_{d}$ is the thickness of the oxide layer. The sensitivity of the ZnO MOSFET was determined experimentally by Ditshego [28] to be approximately 75%. For the new NPG MOSFET, the conductivity $G_{0}$ was measured first without the presence of a chemical analyte and was subsequently measured during the presence of carbon monoxide gas (concentration: 500 ppm). It was found that the conductivity increases to approximately 300% when the carbon monoxide gas is present. (It should be pointed out, however, that the ZnO MOSFET was not tested in the present work, and the data that were relied upon are the only published data [30]).

The parameters listed above can be further understood from the following Table 1:

## 6. Conclusions

A new type of MOSFET with a nanoporous gold (NPG) gate electrode was presented. The new MOSFET was used as a sensor in various electrochemical and bioelectrochemical reactions. Because of the excellent catalytic activity of NPG, the new sensor was highly sensitive to various analytes. As examples, the detection of glucose and of carbon monoxide were tested and reported. By comparison with the older generation of ZnO MOSFETs, we found that this the new MOSFET offers a lower leakage current and a substantially higher sensitivity. References [24,25] describe several additional types of MOSFET that utilize materials such as graphene, MoS_2_, and SnS_2_ for the detection of some specific types of analytes. However, as far as the authors are aware, this paper introduces for the first time a sensor which consists of a MOSFET that is fitted with an NPG gate electrode.

## Figures and Tables

**Table 1:** Main points of comparison between the ZnO MOSFET and the NPG MOSFET.

Parameter	ZnO MOSFET	NPG MOSFET
Deposition of gate electrode	ALD	Sputtering
Leakage current	few hundred nA	1 nA
Sensitivity	75%	300% or higher

## Data Availability

Data associated with this article can be made available upon reasonable request from the authors.
